# Supplementary material for: Ceftriaxone Mediated Synthesized Gold Nanoparticles: A Nano-Therapeutic Tool to Target Bacterial Resistance
Source: Pharmaceutics. 2021 Nov 8;13(11):1896. doi: 10.3390/pharmaceutics13111896 (PMC8622407; doi:10.3390/pharmaceutics13111896)
Supplement: Supplementary file 1 [file pharmaceutics-13-01896-s001.zip › pharmaceutics-1429766-supplementary.pdf]

pharmaceutics

MDPI

Supplementary Materials: Ceftriaxone Mediated Synthesized Gold Nanoparticles: A Nano-Therapeutic Tool to Target Bacterial Resistance

Farhan Alshammari, Bushra Alshammari, Afrasim Moin, Abdulwahab Alamri, Turki Al Hagbani, Ahmed Alobaida, Abu Baker, Salman Khan and Syed Mohd Danish Rizvi

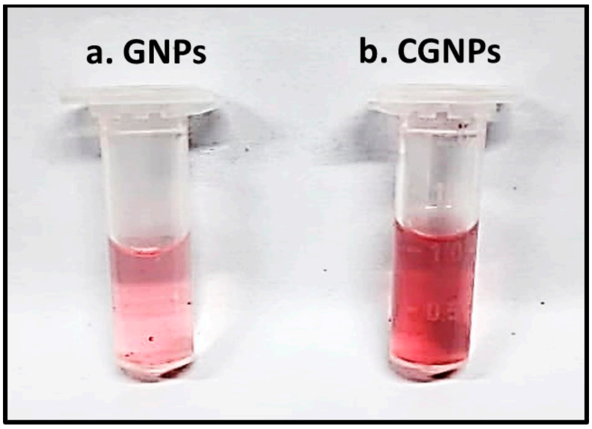

Figure S1. Synthesized gold nanoparticles (**a**) Bromelain mediated synthesized (GNPs) (**b**) Ceftriaxone mediated synthesized (CGNPs).

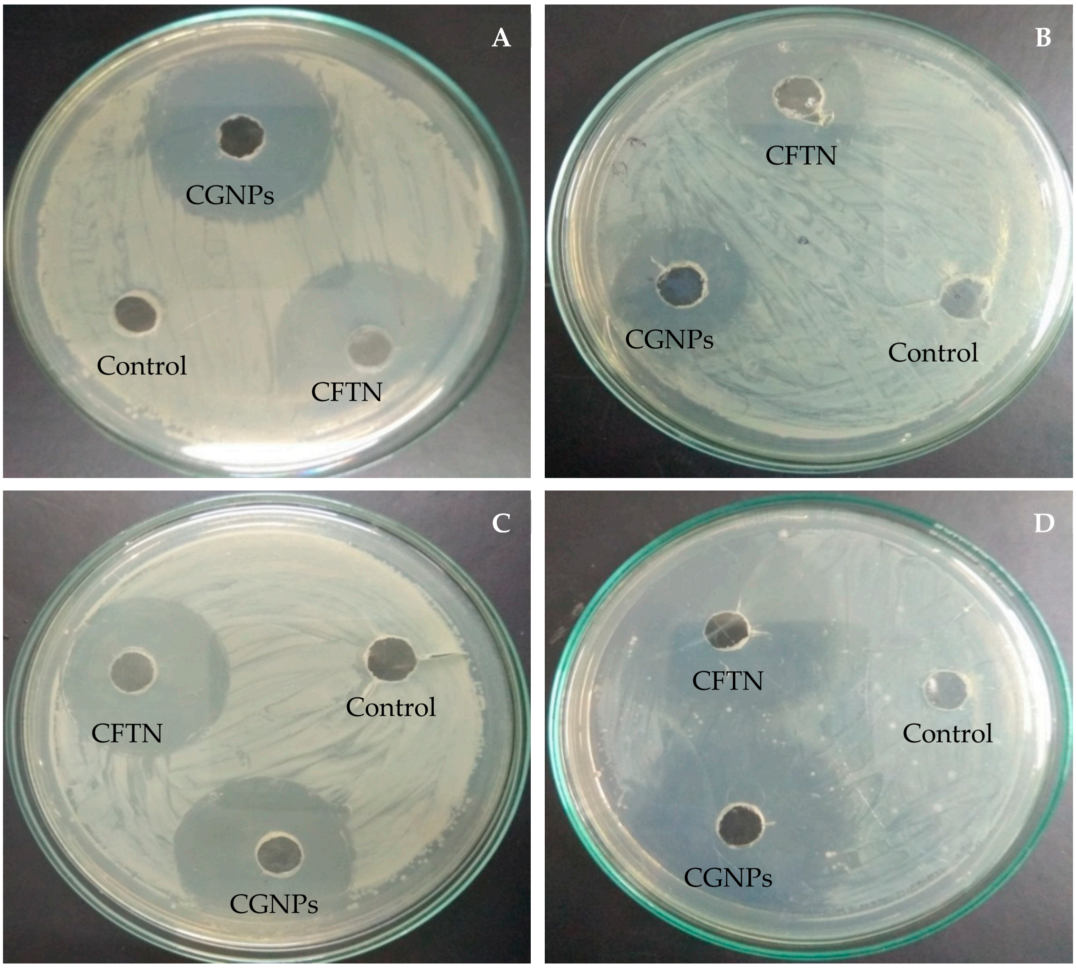

Figure S2. Qualitative assessment of the antibacterial activity of CGNPs and ceftriaxone (CFTN). Müeller-Hinton (MH) agar plates were seeded with standardized suspensions (equivalent to 0.5 McFarland) of (**A**) *Escherichia coli* (**B**) *Staphylococcus aureus*, (**C**) *Salmonella abony*, and (**D**) *Klebsiella pneumonia*. The dilutions of CGNPs 50 μL (200 μg/mL CFTN), CFTN 50 μL (1 mg/mL CFTN), and GNPs 50 μL (negative control) were poured in the wells made in MH plates. After overnight incubation at 37 °C, zones of inhibition around wells of CGNPs and CFTN against all tested bacterial species, in comparison to control, were observed.
